# Activating UCHL1 through the CRISPR activation system promotes cartilage differentiation mediated by HIF‐1α/SOX9

**DOI:** 10.1111/jcmm.70051

**Published:** 2024-09-02

**Authors:** Shanwei Shi, Yang Ge, Qiqian Yan, Shuangquan Wan, Mingfei Li, Maoquan Li

**Affiliations:** ^1^ School of Stomatology, Stomatological Hospital Southern Medical University Guangzhou China; ^2^ Guangdong Academy of Stomatology Guangzhou Guangdong China

**Keywords:** chondrogenesis, CRISPR, hypoxia‐inducible factor‐1α, ubiquitin C‐terminal hydrolase L1

## Abstract

Developing strategies to enhance cartilage differentiation in mesenchymal stem cells and preserve the extracellular matrix is crucial for successful cartilage tissue reconstruction. Hypoxia‐inducible factor‐1α (HIF‐1α) plays a pivotal role in maintaining the extracellular matrix and chondrocyte phenotype, thus serving as a key regulator in chondral tissue engineering strategies. Recent studies have shown that Ubiquitin C‐terminal hydrolase L1 (UCHL1) is involved in the deubiquitylation of HIF‐1α. However, the regulatory role of UCHL1 in chondrogenic differentiation has not been investigated. In the present study, we initially validated the promotive effect of UCHL1 expression on chondrogenesis in adipose‐derived stem cells (ADSCs). Subsequently, a hybrid baculovirus system was designed and employed to utilize three CRISPR activation (CRISPRa) systems, employing dead Cas9 (dCas9) from three distinct bacterial sources to target UCHL1. Then UCHL1 and HIF‐1α inhibitor and siRNA targeting SRY‐box transcription factor 9 (SOX9) were used to block UCHL1, HIF‐1α and SOX9, respectively. Cartilage differentiation and chondrogenesis were measured by qRT‐PCR, immunofluorescence and histological staining. We observed that the CRISPRa system derived from *Staphylococcus aureus* exhibited superior efficiency in activating UCHL1 compared to the commonly used the CRISPRa system derived from *Streptococcus pyogenes*. Furthermore, the duration of activation was extended by utilizing the Cre/loxP‐based hybrid baculovirus. Moreover, our findings show that UCHL1 enhances SOX9 expression by regulating the stability and localization of HIF‐1α, which promotes cartilage production in ADSCs. These findings suggest that activating UCHL1 using the CRISPRa system holds significant potential for applications in cartilage regeneration.

## 1 INTRODUCTION

Repairing damaged cartilage resulting from trauma, congenital malformations or oncological resection poses a significant challenge. Currently available treatment strategies for cartilage injuries in clinical settings are insufficient.^1^ Stem cell‐based strategies employing multipotent mesenchymal stem cells (MSCs) have emerged as a viable approach for promoting cartilage regeneration.^2^ Nevertheless, continuous proliferation of chondrocytes leads to dedifferentiation and loss of phenotypical characteristics, thereby impairing their capacity to form cartilage.^3^ Developing a method to preserve the chondrocyte phenotype is crucial for effective cartilage tissue reconstruction.

When contemplating the application of MSCs in articular cartilage repair, a significant hurdle arises in creating cells that mimic stable chondrocytes, demonstrating resistance to hypertrophy and terminal differentiation, consistent with hyaline articular cartilage characteristics.^4^ Typical in vitro methodologies designed for inducing chondrogenic differentiation in MSCs have proven effective in stimulating the expression of various cartilage‐specific markers such as collagen type II and aggrecan, thereby fostering a chondrocyte‐like phenotype.^5^

Hypoxia‐inducible factor‐1α (HIF‐1α) plays a crucial role in collagen synthesis and modification of chondrocytes during the development of the growth plate under low‐oxygen conditions.^6^, ^7^ Previous research has shown that chondrocytes demonstrate enhanced differentiation under hypoxic conditions, which is associated with HIF‐1α.^8^, ^9^ Suppression of HIF‐1α leads to cartilage catabolism and cell death in the developing limb bud mesenchyme.^10^ Considering the vital role of HIF‐1α in preserving the cartilage extracellular matrix and chondrocyte phenotype,^7^ HIF‐1α serves as the primary regulatory factor for strategies in osteochondral tissue engineering. The signalling cascade involving prolyl hydroxylase 2 (PHD2) and von Hippel–Lindau (VHL) regulates HIF‐1α.^11^ In normoxic conditions, PHD2 hydroxylates the proline residues within the oxygen‐dependent degradation domain (ODDD) of HIF‐1α. Subsequently, the hydroxylated residues undergo ubiquitination by VHL, which contains E3 ubiquitin ligase. This process ultimately causes the rapid proteolysis of HIF‐1α.^12^ In contrast, under hypoxic conditions, HIF‐1α stabilizes and becomes active due to the inactivation of PHD2. Upon nuclear translocation, HIF‐1α, together with transcriptional cofactors, induces the expression of the genes associated with chondrogenesis such as SRY‐box transcription factor 9 (SOX9).^13^ Recent research has demonstrated that activation of UCHL1 leads to the upregulation of HIF‐1α.^14^ Nevertheless, the regulation of chondrogenic differentiation by UCHL1 through HIF‐1α upregulation remains unstudied.

Cas9 is an RNA‐guided endonuclease employed for precise genome editing by targeting the protospacer‐adjacent motif (PAM) and the adjacent protospacer sequence.^15^, ^16^ By utilizing catalytically deactivated dCas9 protein fused with a transcriptional activator (e.g. p65), the CRISPR/Cas9 system can be engineered to act as a precise transcriptional regulator for target genes through CRISPR activation (CRISPRa).^17^ Initially, the first‐generation CRISPRa was developed by combining dCas9 with synthetic p65 or VP64 activation domains, yielding moderate gene expression levels.^18^, ^19^ To achieve more robust gene activation, the system was enhanced by incorporating a tripartite activator named VPR, comprising VP64, p65 and Rta, resulting in the formation of a dCas9‐VPR transactivation module.^20^, ^21^ This module has found extensive application in diverse biological contexts,^22^, ^23^, ^24^ yet its potential in osteochondral tissue engineering has remained largely unexplored.

Through this investigation, we confirmed a significant increase in UCHL1 expression during chondrogenic differentiation of adipose‐derived stem cells (ADSCs) and bone marrow‐derived mesenchymal stem cells (BMSCs). Subsequently, we designed and packaged three dCas9‐VPR complexes, along with their corresponding gRNAs into baculovirus vectors to achieve efficient gene delivery. These complexes effectively induced UCHL1 upregulation for a minimum of 14 days. Additionally, we discovered that UCHL1 regulates the stability of HIF‐1α, which consequently affects the process of SOX9‐mediated chondrogenic differentiation. Therefore, the upregulation of UCHL1 through CRISPRa facilitates the process of cartilage tissue regeneration.

## 2 MATERIALS AND METHODS

### 2.1 Cell culture and reagents

Rat BMSCs (cat. no. CP‐R131) and ADSCs (cat. no. CP‐R147) were obtained from Procell Life Science Technology Co., Ltd. Both cells were cultured in α‐MEM medium (Hyclone, Cytiva) supplemented with 10% fetal bovine serum (FBS), 100 U/mL penicillin and 100 U/mL streptomycin (all from Hyclone, Cytiva) at 37°C with 5% CO_2_. The cells were maintained in a humidified chamber with either 20% O_2_ (normoxia group, NX) or 1% O_2_ (hypoxia group, HX). Insect cells (Sf‐9) and embryonic kidney (293 T) cell lines was described previously.^14^

Stock solutions of LDN57444 (UCHL1 inhibitor, LDN, MilliporeSigma, cat. no. L4170) and KC7F2 (HIF‐1α inhibitor, MilliporeSigma, cat. no. SML1043) were prepared in phosphate buffered saline (PBS) at concentrations of 10 mM and 30 μM, respectively. Small interfering RNA targeting SOX9 (si‐SOX9, 5′‐CGUUUAACCUUCAAGAAU‐3′) and negative control siRNA (si‐NC, si‐Negative Control, 5′‐UGGUUUACAUGUUUUCUGA‐3′) were purchased from RiboBio (Guangzhou, China).

### 2.2 Construction and preparation of baculovirus vectors

The plasmid of pBac‐Sa‐con that expressed *Staphylococcus aureus*‐dCas9‐VP64‐p65‐Rta (Sa‐dCas9‐VPR) but no small guide RNA (sgRNA) was constructed previously.^11^ All primers used for virus construction are listed in Table S1. To construct the plasmid pBac‐Cre, we first replaced the polyhedrin and p10 promoters in pFastBac DUAL vector (Invitrogen, cat. no. 10712024) with the CMV enhancer‐rat EF‐1α (rEF‐1α) promoter from pVITRO1‐neo‐mcs (Invivogen, cat. no. pvitro1‐nmcs) by XhoI/BamHI (Beyotime) treatment to yield pBacE. Then the cDNA of Cre and a woodchuck hepatitis virus post‐transcriptional regulatory element (WPRE) which can enhance the mRNA stability were PCR‐amplified from pENN.AAV.hSyn.Cre.WPRE.hGH (Addgene, cat. no. 105553) and subcloned into pBacE using EcoRI/NotI (Beyotime). The donor plasmid pBac‐Cre and pBac‐Sa‐con were used to generate baculovirus Bac‐Cre and Bac‐Sa‐con using the Bac‐To‐Bac® system (Invitrogen, cat. no. 10359016).

The dCas9 gene from *Streptococcus pyogenes* (Sp‐dCas9) and *Neisseria meningitides* (Nm‐dCas9) were PCR‐amplified from pSLQ2815 (Addgene, cat. no. 84245) and pcDNA3.3‐Nm‐dCas9‐p300Core (Addgene, cat. no. 61365) with a 5′ and 3′ flanking SV40 nuclear localization signal (NLS). During PCR, PacI/MluI sites were appended. Sp‐dCas9 and Nm‐dCas9 were separately cloned into pBac‐Sa‐con to replace Sa‐dCas9 using PacI/MluI (Beyotime), yielding pBac‐Sp‐con and pBac‐Nm‐con, respectively. The sgRNA cassettes of Sa‐dCas9, Sp‐dCas9 and Nm‐dCas9 were synthesized using the sequences on pSLQ2806‐2 (Addgene, cat. no. 84250), pSLQ2804 (Addgene, cat. no. 84258) and pSmart‐Nm‐sgRNA‐BbsI (Addgene, cat. no. 49157), respectively, which contained a human U6 (hU6) promoter, a spacer sequence and a sgRNA scaffold. The spacer sequences targeting UCHL1 with the highest targeting specificity scores (Table S2) were designed using a guide RNA design tool (www.benchling.com). The resultant sgRNA sequences were subcloned into pBac‐Sa‐con, pBac‐Sp‐con and pBac‐Nm‐con, respectively. The resultant pBac‐Sa‐UCHL1, pBac‐Sp‐UCHL1 and pBac‐Nm‐UCHL1 were used to generate baculovirus (BV) vectors Bac‐Sa‐UCHL1, Bac‐Sp‐UCHL1 and Bac‐Nm‐UCHL1 respectively, using the Bac‐To‐Bac® system. The recombinant BV vectors were amplified by infecting Sf‐9 insect cells, titrated by end‐point dilution method.

In the CRISPRa module, sgRNA was expressed under the human U6 promoter, and dCas9‐VPR was expressed under the rat EF‐1α promoter (Figure S1A). We investigated the potential of Sp‐dCas9, Sa‐dCas9 and Nm‐dCas9 for activating UCHL1 in ADSCs and BMSCs. This was done by generating three BV vectors that co‐express the effector and sgRNA, targeting distinct positions downstream of the transcription start site of UCHL1. Bac‐Sa‐UCHL1 expressed Sa‐dCas9‐VPR along with its associated sgRNA (−13). On the other hand, Bac‐Sp‐UCHL1 and Bac‐Nm‐UCHL1 expressed Sp‐dCas9‐VPR, sgRNA (−189), and Nm‐dCas9‐VPR, sgRNA (−258), respectively. As a control, we generated Bac‐Sa‐con, which expressed Sa‐dCas9‐VPR without any associated sgRNA (Figure S1 B).

### 2.3 Baculovirus transduction

BMSCs and ADSCs were seeded in 6‐well plates at a density of 1 × 10^5^ cells/well for qRT‐PCR analysis, and in 10‐cm dishes at a density of 1 × 10^6^ cells/dish for immunofluorescence (IF) staining and scaffold seeding purposes. For transduction, cells cultured overnight were washed twice with PBS before being transduced either with Bac‐Cre alone or co‐transduced with Bac‐Cre along with additional Bac‐Sa‐con, Bac‐Sa‐UCHL1, Bac‐Sp‐UCHL1 or Bac‐Nm‐UCHL1. Depending on the multiplicity of infection (MOI, pfu/cell) and virus titre, an appropriate volume of virus supernatant was mixed with NaHCO_3_‐free α‐MEM at a volumetric ratio of 1:4. This mixture, totaling 0.5 mL for 6‐well plates and 2 mL for 10‐cm dishes, was subsequently added to the cells. The cells were gently shaken on a rocking plate at 25°C for 6 h. Afterward, the medium was replaced with α‐MEM medium supplemented with 3 mM sodium butyrate (Sigma), and the cells continued to be cultured. At 1 day post transduction (dpt), the medium was replaced with either fresh α‐MEM or chondroinductive medium. The chondroinductive medium consisted of α‐MEM supplemented with 1% FBS, 100 U/mL penicillin, 100 U/mL streptomycin, 1% insulin‐transferrin‐sodium selenite (ITS) liquid media supplement (Corning, cat. no. 354352), 10 nM dexamethasone (Sigma, cat. no. 50‐02‐2), and 10 ng/mL transforming growth factor‐β1 (TGF‐β1, PeproTech, cat. no. 100‐21). The fresh chondroinductive medium was replaced every 2–3 days until subsequent in vitro analyses were conducted. Micromass culturing was conducted according to the established protocol.^8^ In summary, cells were resuspended in α‐MEM at a density of 2 × 10^7^ cells/mL. Then, a 20 μL cell suspension was placed in the center of a 24‐well plate and incubated for 3 h. Subsequently, chondrogenic differentiation medium was added and refreshed every 2–3 days for 14 days. For the 3D culture experiments, the cells were harvested and seeded onto gelatine scaffolds. The chondrogenic differentiation medium was added and replaced every 2–3 days for 14 days.

### 2.4 Quantitative real‐time reverse transcription PCR (qRT‐PCR)

The RNA extraction kit (ESscience, cat. no. RN001) was used to isolate total RNA from cells. The concentration and quality of the total RNA samples were assessed using Nanodrop2000 (Thermo Fisher Scientific Inc). Around 1000 ng of RNA was reverse‐transcribed to cDNA using the PrimeScript™ RT Master Mix (Takara, cat. no. RR047A). Then, quantitative real‐time PCR was performed in triplicate using gene‐specific primers (Table S3) and the PrimeScript RT‐PCR kit (Takara, cat. no. RR820A). The data were analysed by calculating 2^−ΔΔCt^, normalizing against GAPDH or 18S, and referencing the control cells (transduced by Bac‐Cre) to yield the relative expression levels.

### 2.5 Alcian blue staining

To perform sulphated glycosaminoglycan (GAG) staining, cells were fixed in 4% (w/v) paraformaldehyde at 25°C for 15 min. They were then washed twice with PBS and stained with a 0.5% Alcian blue solution (Sigma, cat. no. B8438) at 25°C for 30 min. After staining, the cells were washed with distilled water and captured under a light microscope (Axio Observer Z1, Zeiss GmbH).

### 2.6 Preparation of ADSC/scaffold constructs and engineered cartilage

Gelatine sponge scaffolds were fabricated by cutting the gelatine sponge (Ethicon, cat. no. MS0002) into disks with a diameter of approximately 6 mm and a thickness of approximately 1 mm. These disks were then immersed in PBS for 30 min. The ADSCs, which were cultured in 10 cm diameter dishes, were transduced as previously described. At 1 dpt, the cells were trypsinized and then seeded onto the gelatine scaffold at a density of 5 × 10^6^ cells/scaffold. Subsequently, they were incubated for 4 h in a 37°C incubator. The ADSCs/scaffold constructs were initially cultured with chondroinductive medium overnight. Subsequently, the culture was continued using the same chondroinductive medium, with a half medium exchange performed every 3 days for 14 days to promote in vitro cartilage formation.

### 2.7 Qualitative and quantitative analysis of engineered cartilage

At 8 dpt, the ADSCs/scaffold constructs were frozen at −80°C overnight and then crushed using a disposable pestle. For GAG content detection, the crushed constructs were incubated with a papain solution (Sigma‐Aldrich) at 65°C for 4 h. For GAG content detection, the crushed constructs were incubated with a papain solution (Sigma‐Aldrich, cat. no. 9001‐73‐4) at 65°C for 4 h. After centrifuging half of the papain‐digested sample (10,000 × *g*, 5 min), the GAG in the supernatant was assayed using the Blyscan™ Sulfated Glycosaminoglycan Assay Kit (Biocolor, cat. no. B1000). The other half was collected for total DNA content analysis using the Quant‐iTTM Picogreen® dsDNA Reagent and Kits (Invitrogen, cat. no. P7589). The absorbance value of the treated samples was measured at 620 nm. For the determination of collagen type II (Col II), each crushed construct was homogenized in a buffer containing 0.05 M acetic acid and 0.5 M sodium chloride for 1 hour. Subsequently, it was digested with pepsin at 4°C overnight and further digested with elastase (Sangon biotech, cat. no. 39445–21‐1) at 4°C overnight. After centrifuging half of the sample (10,000 × *g*, 5 min), the supernatant was then measured using an ELISA‐based Type II Collagen Detection Kit (Chondrex, cat. no. 6018). The optical density (OD) of the treated samples was assayed at 450 nm. The total GAG and Col II contents were normalized to the total DNA content of the corresponding construct.

At 14 dpt, the ADSCs/scaffold constructs were imaged, then washed three times with PBS, and subsequently fixed in 4% paraformaldehyde overnight at a temperature of 25°C. Following fixation, the constructs were dehydrated, embedded in paraffin, and sliced into 4 μm thick sections. The sections were stained with haematoxylin and eosin to visualize cell morphology. Alternatively, they were stained with a 0.5% Alcian blue solution to detect sulphated glycosaminoglycans.

### 2.8 Cell proliferation

The CCK‐8 (Cell Counting Kit‐8) cytotoxicity assay (DOJNDO, cat. no. CK04‐11) was used to determine the optimal concentration of HIF‐1α inhibitor. Once ADSCs were seeded into 96‐well plates, α‐MEM media with different concentrations (0, 10, 20, 30, 40 or 50 μM) of HIF‐1a inhibitor were added for 1, 3, 5 and 7 days. Subsequently, 10 μL of CCK‐8 reagent was added to each well, and the plates were incubated at 37°C for 2 h. The absorbance of the supernatant was measured at 450 nm using a microplate reader (Thermo Scientific Multiskan Spectrum).

### 2.9 TUNEL staining

The level of damaged DNA was assessed using the TUNEL cell apoptosis detection kit (Beyotime, cat. no. C1086). Cells from various experimental groups were fixed and subjected to staining with the TUNEL test solution for 30 min at 37°C, following the manufacturer's instructions. Three randomly chosen fields were captured to count the number of TUNEL‐positive cells.

### 2.10 IF staining

Cells transduced with Bac‐Cre (Cre group), Bac‐Cre/Bac‐Sa‐con (Sa‐con group), or Bac‐Cre/Bac‐Sa‐UCHL1 (Sa‐UCHL1 group) and treated with si‐SOX9 or si‐NC were subjected to immunofluorescence (IF) staining using standard procedures.^14^ Additionally, to investigate the effects of hypoxia on the expression level of SOX9, cells were cultured under normoxia (20%) or hypoxia conditions for 24 h. To confirm the influence of HIF‐1α on SOX9, cells were treated with HIF‐1α inhibitor for 4 h. The primary antibodies used were rabbit anti‐HIF‐1α (1:1000, Cell Signaling Technology, cat. no. 36169) and rabbit anti‐SOX9 (1:1000, Cell Signaling Technology, cat. no. 82630). The secondary antibody used was Alexa Fluor® 488‐conjugated goat anti‐rabbit IgG (1:1000, Abcam, cat. no. ab150077). Images were acquired using a laser scanning confocal microscope (Zeiss GmbH, cat. no. LSM780).

### 2.11 Transient transfection and luciferase assays

The SOX9 promoter region was amplified by PCR using genomic DNA from 293T cells. Subsequently, the amplified fragment was inserted into the HindIII/BglII sites of the pGL4 vector to create the pGL4‐SOX9 parental construct (Table S4). The pGL4‐SOX9 mutant construct was generated through site‐directed mutagenesis of the pGL4‐SOX9 vector. The reporter constructs were co‐transfected into 293T cells along with various expression vectors and internal control plasmids under either normoxic or hypoxic conditions, following a previously described protocol.^9^ The cells were harvested and assayed using a luciferase reporter assay system (Promega, cat. no. E1910) according to the manufacturer's instructions.

### 2.12 Chromatin immunoprecipitation (ChIP)‐PCR

ChIP assays were conducted following the protocol of the SimpleChIP® Enzymatic Chromatin IP kit (Cell Signaling Technology, cat. no. 9002). Approximately 1 × 10^7^ ADSCs cultured in a 15 cm dish were cross‐linked by incubating with formaldehyde (final concentration of 1%) at 25°C for 10 min and subsequently quenched by incubating with 500 μL of 2.5 M glycine for 5 min. The cross‐linked cells were washed three times with PBS on ice, harvested with 1 mL of PBS, and pelleted at 3000 rpm for 5 min. Subsequently, the cells were incubated and resuspended successively in buffers A and B. DNA was sheared using 5 μL of micrococcal nuclease at 37°C for 20 min. Digestion was then halted, and the cells were sonicated to disrupt the nuclei. Centrifugation was performed to collect the extracts, and the resulting supernatants were transferred to new tubes. ChIP assays were conducted employing HIF‐1α antibody (Cell Signaling Technology, cat. no. 36169), with Normal H3 and IgG serving as negative controls. DNA‐protein‐antibody complexes were incubated with protein A beads, followed by dilution of the complexes using a dilution buffer, with a 1% fraction removed as input. Additional samples were treated with specific antibodies based on the experimental groups and incubated overnight at 4°C. Subsequently, the complexes were washed, and the DNA was eluted in elution buffer containing proteinase K overnight at 65°C. The DNA fragment was purified and subjected to qRT‐PCR analysis using the following primers: forward primer 5′‐GACTCCAGGCGCAGAAGCCC‐3′, reverse primer 5′‐CCGGGACTTCGCTGGCGTTT‐3′. The experiment was conducted in triplicate and repeated thrice.

### 2.13 Statistical analysis

The data were expressed as means ± standard deviations (SD) from a minimum of three independent experiments. After performing Shapiro–Wilk tests to assess normality and Levene's test to evaluate the equality of variances, we employed a paired Student's *t*‐test to compare the differences between the control and test groups. For the analysis of differences in the CCK‐8 assays and quantitative measurements of HIF‐1α and SOX9, we employed one‐ and two‐way ANOVA followed by a SNK post hoc test. Statistical significance was defined as *p* value <0.05.

## 3 RESULTS

### 3.1 UCHL1 expression in ADSCs and BMSCs promoted chondrogenic differentiation

**FIGURE 1:**
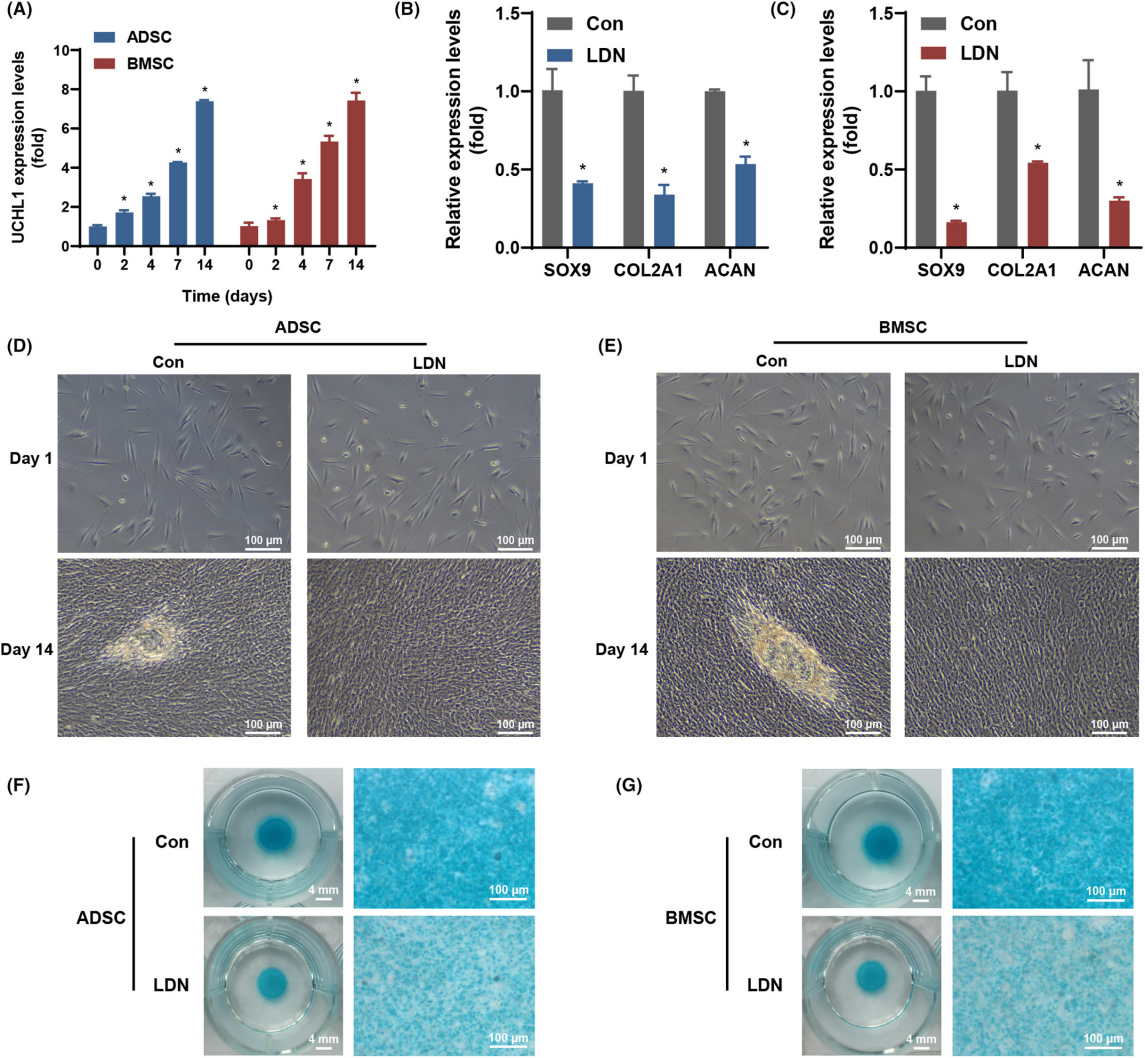
UCHL1 expression in adipose‐derived stem cells (ADSCs) and bone marrow‐derived mesenchymal stem cellS (BMSCs) promoted chondrogenic differentiation. (A) The mRNA expression level of UCHL1 was analysed during chondrogenic differentiation in ADSCs and BMSCs. (B, C) The mRNA expression levels of SOX9, COL2A1, and ACAN were assessed in ADSCs (B) and BMSCs (C) after treatment with UCHL1 inhibitor for 7 days. (D, E) The effects of LDN‐mediated inhibition of UCHL1 on cell morphology were assessed during chondrogenic differentiation in ADSCs (D) and BMSCs (E) at 1 and 14 days. (F, G) The effects of UCHL1 inhibitor‐mediated inhibition of UCHL1 on sulphated GAG accumulation, stained by Alcian blue, were assessed during chondrogenic differentiation in ADSCs (F) and BMSCs (G) at 14 days (data are presented as means ± SD, significant differences are presented as *p < 0.05).

ADSCs and BMSCs were cultured in chondroinductive medium, and the expression of UCHL1 was analysed by qRT‐PCR. The results demonstrated that UCHL1 expression increased over time in both ADSCs and BMSCs (Figure 1A), indicating its involvement in the chondrogenesis of mesenchymal stem cells. In order to investigate the function of UCHL1 in chondrogenesis of mesenchymal stem cells, we utilized LDN, a small molecule inhibitor of UCHL1, to suppress its expression in ADSCs and BMSCs. Chondrogenic marker genes, including SOX9 (0.4‐fold at ASC and 0.2‐fold at BMSC), collagen type II alpha 1 (COL2A1, 0.3‐fold at ASC and 0.5‐fold at BMSC) and aggrecan (ACAN, 0.5‐fold at ASC and 0.3‐fold at BMSC), were assessed at 7 days through qRT‐PCR analysis in LDN‐treated samples (Figure 1B,C). After 14 days, the LDN group did not display significant indications of cell differentiation (Figure 1D,E) or accumulate a substantial amount of sulphated GAG, a cartilage‐specific extracellular matrix, compared to the Con group (Figure 1 F,G). This observation supports the notion that suppression of UCHL1 leads to lower levels of cell differentiation and the cartilage‐specific extracellular matrix of ADSCs and BMSCs in vitro.

### 3.2 UCHL1 activation by dCas9‐VPR stimulated chondrogenesis

To induce UCHL1 upregulation, ADSCs and BMSCs were transduced with Bac‐Cre alone (NC group) or co‐transduced with Bac‐Cre and additional vectors such as Bac‐Sa‐con, Bac‐Sa‐UCHL1, Bac‐Sp‐UCHL1 or Bac‐Nm‐UCHL1. For example, the co‐transduction of Bac‐Cre and Bac‐Sa‐UCHL1 was designated as the Sa‐UCHL1 group. The expression of UCHL1 was analysed by qRT‐PCR at 3 dpt. Sa‐UCHL1 and Sp‐UCHL1 resulted in significant upregulation of UCHL1 compared to the Sa‐con group. However, Nm‐UCHL1 group showed only slight activation of UCHL1 in ADSCs, which was not statistically significant (Figure S1C). Similar findings were observed in BMSCs as well (Figure S1D). Considering that Sa‐UCHL1 exhibited the most pronounced UCHL1 activation, we employed Bac‐Cre/Bac‐Sa‐UCHL1 for further experiments. To ascertain whether the CRISPRa system enhanced UCHL1 activation through the formation of episomal minicircles that prolonged its effect, we assessed the UCHL1 level using qRT‐PCR for 14 days. In both ADSCs and BMSCs, the Sa‐UCHL1 group exhibited consistently elevated levels of UCHL1 at all time points, indicating that the CRISPRa system activated UCHL1 expression for a minimum of 14 days (Figure S1E,F).

**FIGURE 2:**
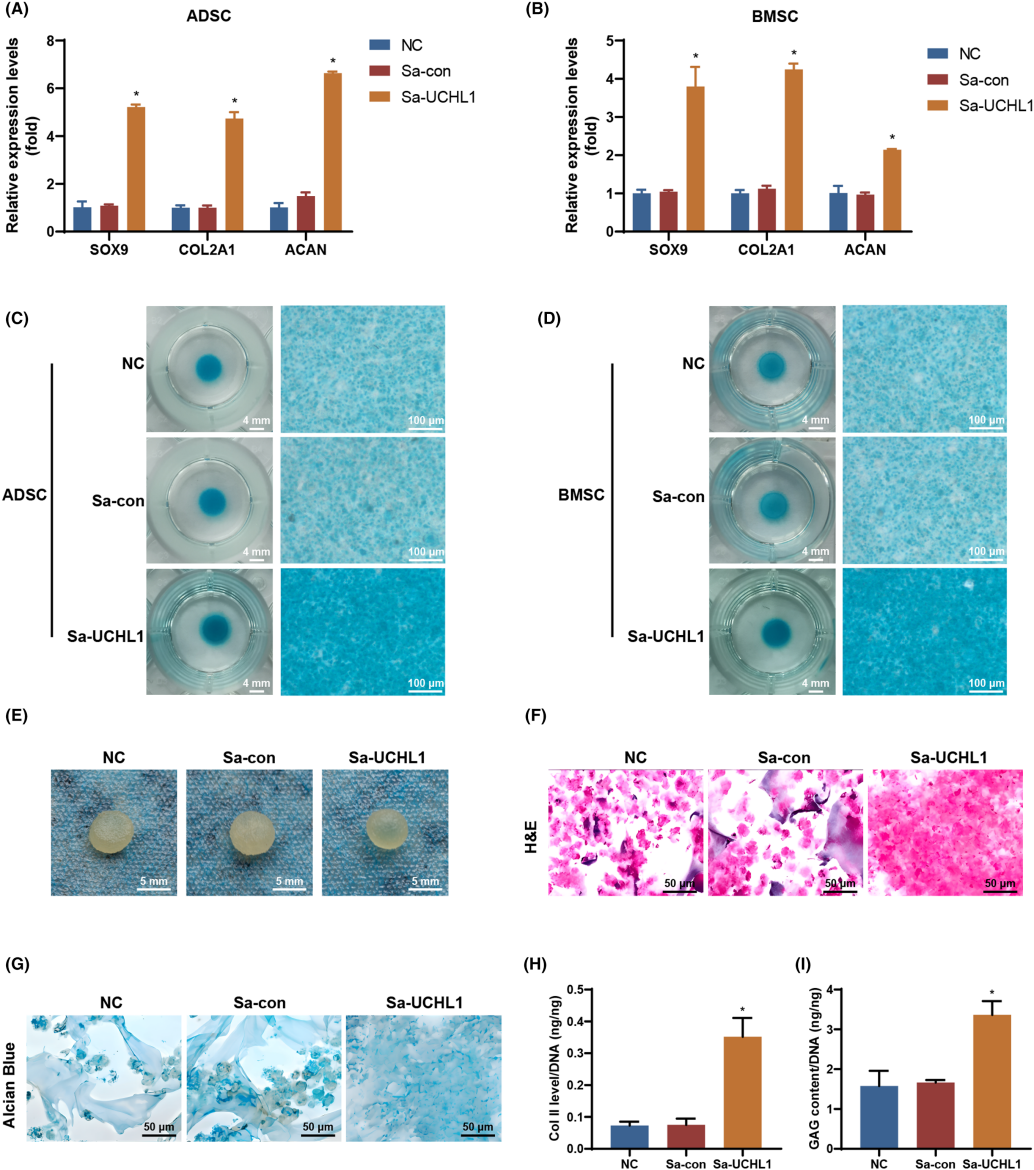
UCHL1 activation by dCas9‐VPR stimulated chondrogenesis. (A, B) The mRNA expression levels of SOX9, COL2A1, and ACAN in adipose‐derived stem cells (ADSCs) (A) and bone marrow‐derived mesenchymal stem cellS (BMSCs) (B) transduced with Bac‐Cre (NC group), Bac‐Cre/Bac‐Sa‐con (Sa‐con group) or Bac‐Cre/Bac‐Sa‐UCHL1 (Sa‐UCHL1 group) at 7 dpt. (C, D) The effects of UCHL1 activation using the CRISPRa system on sulphated GAG accumulation, as assessed by Alcian blue staining, were investigated in ADSCs (C) and BMSCs (D). (E‐G) Gross appearance (E), haematoxylin and eosin staining (F), and Alcian blue staining (G) were assessed in the NC, Sa‐con, and Sa‐UCHL1 groups. (H) Quantitative analysis of Col II levels, normalized to the total DNA content, was performed in the NC, Sa‐con, and Sa‐UCHL1 groups. (I) Quantitative analysis was conducted to measure the GAG content, normalized to the total DNA content, in the NC, Sa‐con, and Sa‐UCHL1 groups (NC, negative control, data are presented as means ± SD, significant differences are presented as *p < 0.05).

After activating UCHL1 using dCas9‐VPR, we evaluated the chondrogenic differentiation of ADSCs and BMSCs. ADSCs and BMSCs were transduced with Bac‐Cre (Negative Control group, NC group), Bac‐Cre/Bac‐Sa‐con (Sa‐con group) or Bac‐Cre/Bac‐Sa‐UCHL1 (Sa‐UCHL1 group) and then cultured in chondroinduction medium. At 7 dpt, there was no significant statistical difference in the expression of SOX9, COL2A1 and ACAN between the Sa‐con group and NC group in ADSCs and BMSCs. However, the Sa‐UCHL1 group showed significantly higher expression levels of SOX9, COL2A1 and ACAN compared to the NC group (Figure 2A,B). Alcian blue staining conducted at 14 dpt revealed that the Sa‐UCHL1 group exhibited a greater accumulation of GAG in ADSCs and BMSCs compared to the NC and Sa‐con groups (Figure 2C,D). These results provide confirmation that UCHL1 activation through dCas9‐VPR promotes chondrogenic differentiation in ADSCs and BMSCs.

In order to verify if UCHL1 activation promotes cartilage formation, we seeded the aforementioned ADSCs into gelatine scaffolds (5 × 10^6^ cells/scaffold, The blank scaffold was shown in Figure S2) and subsequently cultured the cell/scaffold constructs in chondroinductive medium. At 14 dpt, the Sa‐UCHL1 group exhibited the deposition of glassy extracellular matrix‐like materials with a whitish appearance, while the NC and Sa‐con groups maintained irregular shapes with rough surfaces (Figure 2E). Consistently, histological examination with haematoxylin and eosin staining (Figure 2F) and Alcian blue staining (Figure 2G) demonstrated that the cells in the Sa‐UCHL1 group exhibited high condensation and accumulated a higher amount of cartilage‐specific GAG compared to the NC and Sa‐con groups. Additional verification was provided by Col II‐specific ELISA (Figure 2H) and quantification of GAG analysis (Figure 2I), demonstrating that the Sa‐UCHL1 group deposited higher levels of Collagen II and GAG compared to the NC and Sa‐con groups. These findings illustrate that UCHL1 activation through dCas9‐VPR promotes the formation of engineered cartilage in a three‐dimensional (3D) environment.

After activating UCHL1 with dCas9‐VPR, DNA damage was assessed using TUNEL staining. The findings indicated a decrease in DNA damage within the Sa‐UCHL1 group as opposed to both the NC and con groups (Figure S3).

### 3.3 HIF‐1α is the target of UCHL1 promoting chondrogenic differentiation

**FIGURE 3:**
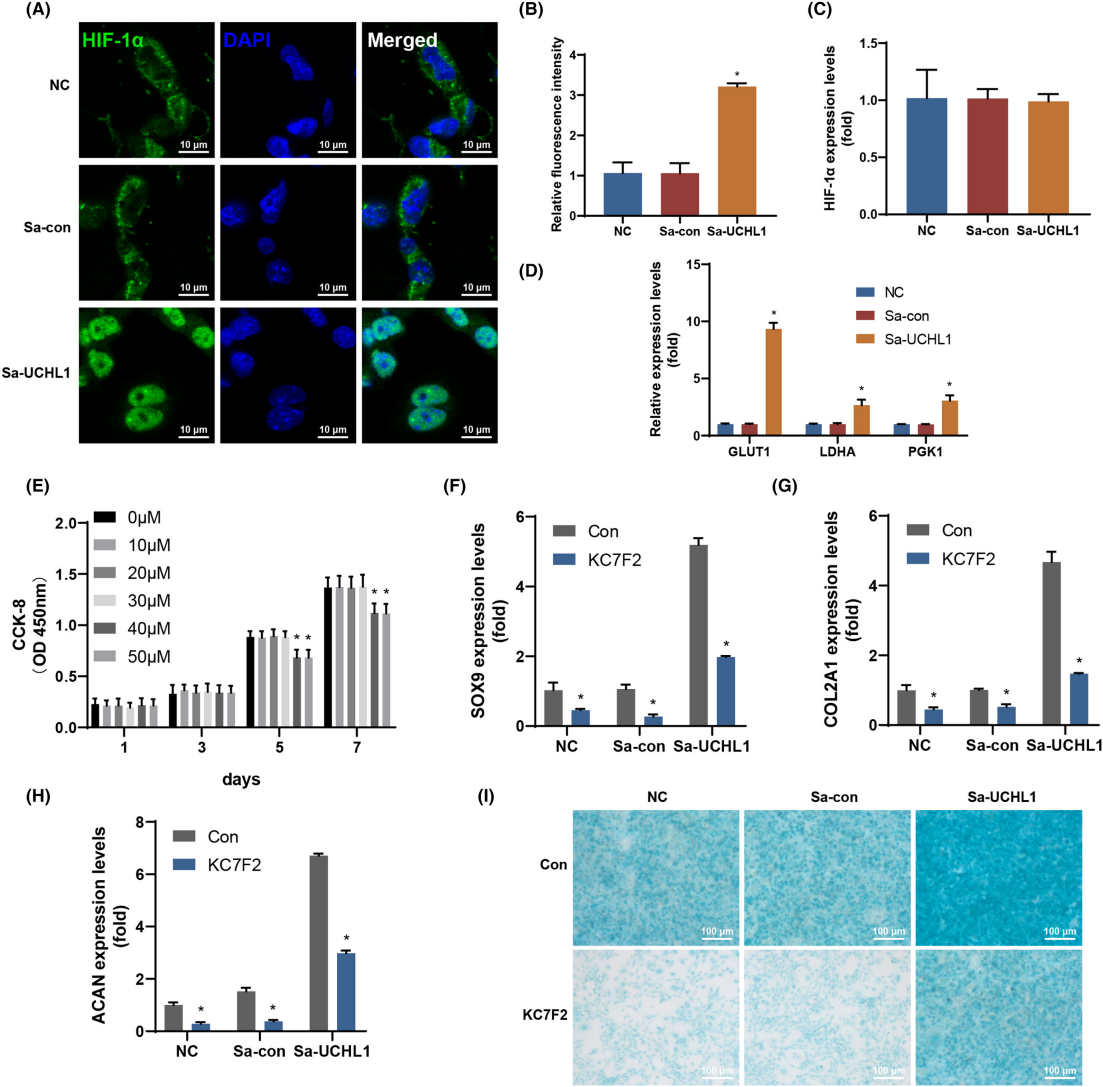
HIF‐1α is the target of UCHL1 promoting chondrogenic differentiation. (A, B) Immunofluorescent staining images of HIF‐1α (A) and the corresponding quantification (B) were performed in the NC, Sa‐con, and Sa‐UCHL1 groups. (C) The mRNA expression level of HIF‐1α was measured in the NC, Sa‐con, and Sa‐UCHL1 groups. (D) The mRNA expression levels of glucose transporter 1 (GLUT1), lactate dehydrogenase A (LDHA) and phosphoglycerate kinase 1 (PGK1) were assessed in the NC, Sa‐con, and Sa‐UCHL1 groups. (E) CCK‐8 assays were performed on cells treated with HIF‐1α inhibitor at indicated concentrations. (F–H) The mRNA expression levels of SOX9 (F), COL2A1 (G), and ACAN (H) were evaluated after treatment with HIF‐1α inhibitor in the NC, Sa‐con, and Sa‐UCHL1 groups. (I) Alcian blue staining was conducted in ADSCs following treatment with HIF‐1α inhibitor in the NC, Sa‐con, and Sa‐UCHL1 groups (NC, negative control, data are presented as means ± SD, significant differences are presented as *p < 0.05).

ADSCs transduced with Bac‐Cre, Bac‐Cre/Bac‐Sa‐con or Bac‐Cre/Bac‐Sa‐UCHL1 were used to measure the protein and mRNA levels of HIF‐1α. Immunofluorescent staining results illustrate a significant increase in HIF‐1α protein expression in the Sa‐UCHL1 group (Figure 3A,B). The mRNA level of HIF‐1α showed no statistical difference among the three groups (Figure 3C), indicating that UCHL1 directly regulates the protein function of HIF‐1α without affecting its transcription.

In the Sa‐UCHL1 group, there was a significantly higher expression of HIF‐1α‐regulated target genes, including glucose transporter 1 (GLUT1), lactate dehydrogenase A (LDHA) and phosphoglycerate kinase 1 (PGK1), compared to the other two groups (Figure 3D).

In order to investigate the impact of HIF‐1α on UCHL1‐induced chondrogenic differentiation, ADSCs were treated with KC7F2, a selective inhibitor of HIF‐1α translation. CCK‐8 assay results indicated that HIF‐1α inhibitor had no cytotoxic effects on chondrocytes at concentrations of 0, 10, 20 and 30 μM after 1, 3, 5 and 7 days of treatment (Figure 3E). However, cell proliferation was significantly inhibited after 5 days of treatment with 40 and 50 μM concentrations. Consequently, a concentration of 30 μM HIF‐1α inhibitor was utilized in subsequent experiments. Subsequently, SOX9, COL2A1 and ACAN levels were assessed using qRT‐PCR, and the results demonstrated a significant decrease in the expression of SOX9, COL2A1 and ACAN in ADSCs treated with HIF‐1α inhibitor for 7 days (Figure 3F–H). Additionally, inhibiting HIF‐1α with HIF‐1α inhibitor led to the suppression of SOX9, COL2A1 and ACAN accumulation induced by UCHL1. After 14 days, the HIF‐1α inhibitor‐treated group showed a lower accumulation of sulphated GAG compared to the control group (Figure 3I), indicating that suppression of HIF‐1α abolished the UCHL1‐induced elevation in sulphated GAG levels. These results indicated that HIF‐1α mediates UCHL1‐promoted chondrogenic differentiation.

### 3.4 HIF‐1α activated SOX9 through the hypoxia‐response element (HRE)

**FIGURE 4:**
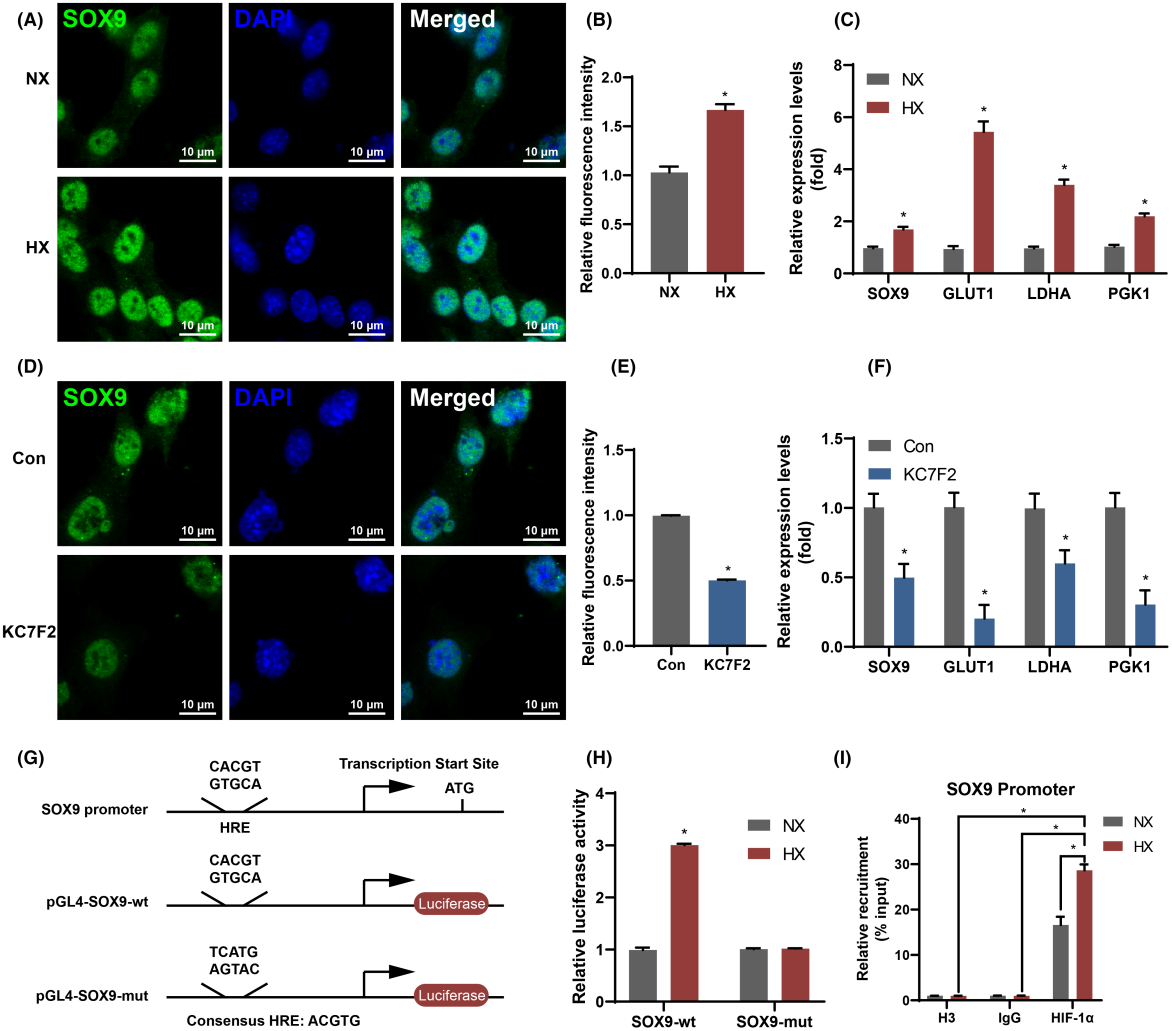
HIF‐1α activated SOX9 through HRE. (A, B) Immunofluorescent staining images of SOX9 (A) and the corresponding quantification (B) were obtained under normoxia or hypoxia conditions. (C) The mRNA expression levels of SOX9, glucose transporter 1 (GLUT1), lactate dehydrogenase A (LDHA) and phosphoglycerate kinase 1 (PGK1) under normoxia or hypoxia conditions. (D, E) Immunofluorescent staining images of SOX9 (D) and the corresponding quantification (E) were performed following treatment with HIF‐1α inhibitor. (F) The mRNA expression levels of SOX9, GLUT1, LDHA, and PGK1 were evaluated after treatment with HIF‐1α inhibitor. (G) Schematic representation depicts the promoter region of SOX9 and the reporter constructs employed in HIF‐1α transfection experiments. The constructs included wild‐type (SOX9‐wt) or mutant (SOX9‐mut) HRE. (H) Activation of either SOX9‐wt or SOX9‐mut was observed following co‐transfection of HIF‐1α under normoxic or hypoxic conditions. (I) ChIP‐PCR with HIF‐1α‐antibody in adipose‐derived stem cells (ADSCs) cultured under normoxic or hypoxic conditions (data are presented as means ± SD, significant differences are presented as *p < 0.05).

We further investigated the regulation of SOX9 expression by HIF‐1α using qRT‐PCR and immunofluorescence analysis. Additionally, we examined the expression levels of downstream targets of HIF‐1α, including GLUT1, LDHA and PGK1. Hypoxic conditions led to upregulation of the protein level of SOX9 and the mRNA levels of SOX9 and the downstream targets of HIF‐1α (Figure 4A–C). However, the above results were reversed when cells were treated with HIF‐1α inhibitor (Figure 4D–F).

In order to clarify the underlying mechanism by which HIF‐1α regulates SOX9, we identified a putative HRE in the proximal promoter region of the SOX9 gene (Figure 4G); detailed sequence information can be found in Table S4. Transient transfections were conducted to assess the activation of the SOX9 promoter in response to hypoxia. Interestingly, hypoxic conditions led to a substantial three‐fold increase in SOX9 promoter activity. Introducing site‐directed mutations into the putative HRE sequence on the SOX9 promoter abolished its activation under hypoxic conditions (Figure 4H). Moreover, ChIP assays yielded evidence supporting the direct interaction between HIF‐1α and the HRE on the SOX9 promoter. PCR amplification of the ChIP samples using an anti‐HIF‐1α antibody showed an increase under hypoxic conditions (Figure 4I).

### 3.5 SOX9 is critical for HIF‐1α‐ and hypoxia‐induced chondrogenesis

**FIGURE 5:**
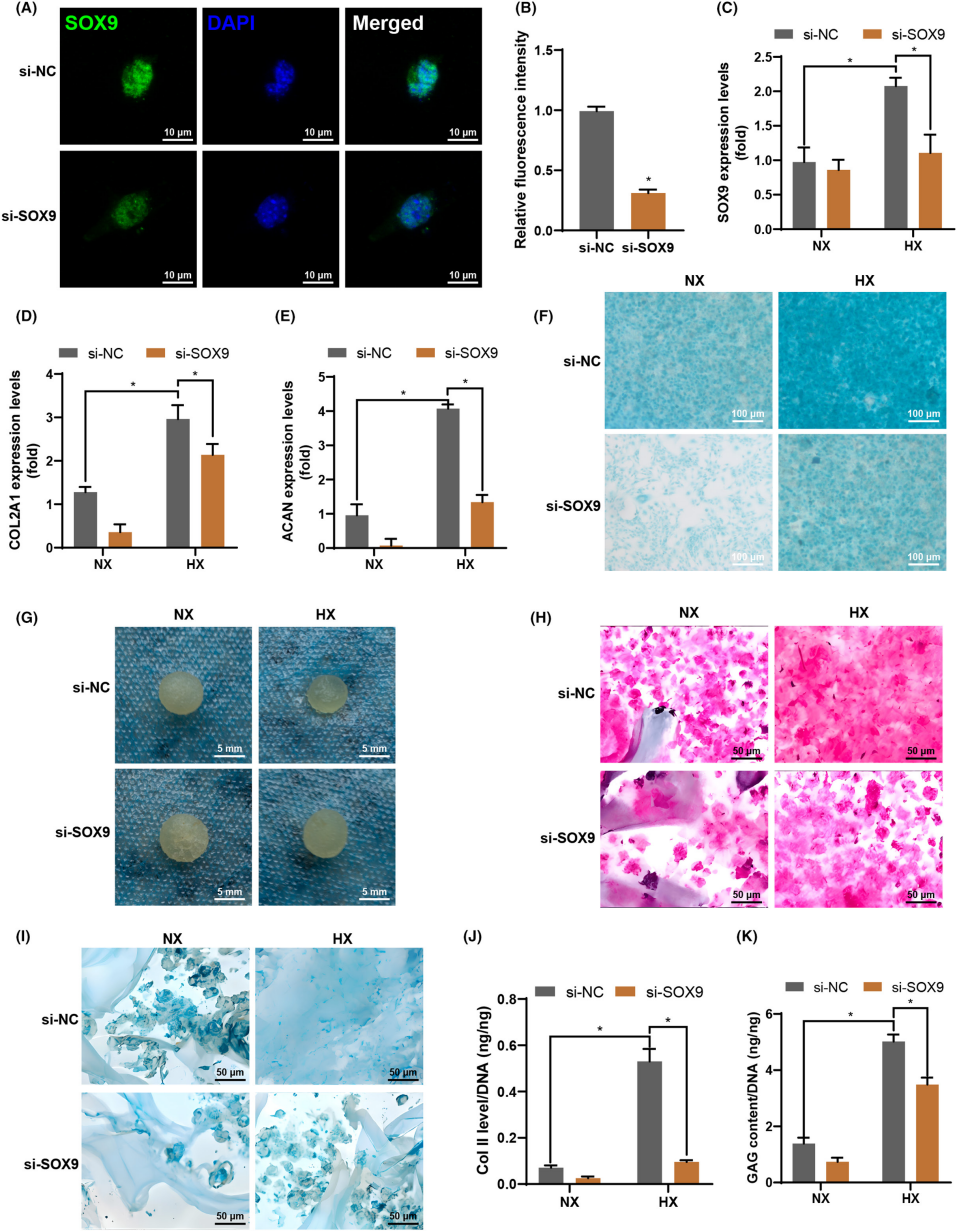
SOX9 is critical for HIF‐1α‐ and hypoxia‐induced chondrogenesis. (A, B) Immunofluorescent staining images of SOX9 (A) and the corresponding quantification (B) were performed in si‐SOX9 and si‐NC groups. (C–E) The mRNA expression levels of SOX9 (C), COL2A1 (D), and ACAN (E) were evaluated after treatment with si‐SOX9 or si‐NC under normoxic or hypoxic conditions. (F) Alcian blue staining was conducted in si‐SOX9 and si‐NC groups. (G–I) Gross appearance (G), haematoxylin and eosin staining (H), and alcian blue staining (I) were assessed after treatment with si‐SOX9 or si‐NC under normoxic or hypoxic conditions. (J) Quantitative analysis of Col II normalized to total DNA content after treatment with si‐SOX9 or si‐NC under normoxic or hypoxic conditions. (K) Quantitative analysis of GAG content normalized to total DNA content after treatment with si‐SOX9 or si‐NC under normoxic or hypoxic conditions (si‐NC, si‐negative control, data are presented as means ± SD, significant differences are presented as *p < 0.05).

To demonstrate whether SOX9 is required for HIF‐1α and hypoxia‐induced chondrocyte differentiation and chondrogenesis, we utilized siRNA to repress the expression of SOX9 (Figure 5A,B). The downregulation of SOX9 resulted in a reduction of the hypoxia‐induced upregulation of SOX9, COL2A1 and ACAN expression levels (Figure 5C–E). Alcian blue staining conducted at 14 dpt revealed that siRNA‐mediated inhibition of SOX9 led to the cessation of hypoxia‐induced GAG accumulation (Figure 5F). The cells were seeded into gelatine scaffolds and cultured in a chondroinductive medium. After 14 dpt, the cells/scaffold constructs exhibited the deposition of ECM‐like materials with a glassy appearance under hypoxic conditions, which became rough upon the inhibition of SOX9 by siRNA (Figure 5G). Haematoxylin and eosin staining (Figure 5H) and Alcian blue staining (Figure 5I) demonstrated that the reduction of SOX9 resulted in a decrease in the accumulation of hypoxia‐induced cartilage‐specific GAG. This observed trend was corroborated by Collagen II‐specific ELISA (Figure 5J) and quantitative GAG analysis (Figure 5K), which provided consistent findings with the haematoxylin and eosin and Alcian blue staining. These findings demonstrated that HIF‐1α and hypoxia‐induced chondrocyte differentiation and chondrogenesis is mediated by SOX9.

## 4 DISCUSSION

The ubiquitin‐proteasome system has been implicated in various human diseases, including cancer,^25^ cardiovascular diseases^26^ and neurodegenerative disorders.^27^ Deubiquitinating enzymes are responsible for the removal of ubiquitin from its target proteins.^28^ There are a total of five subfamilies of DUBs, namely the Ub C‐terminal hydrolases, ovarian tumour domain‐containing proteins, Ub‐specific proteases, Jab1/MPN domain‐associated metalloisopeptidase‐containing proteins and Josephin‐domain‐containing proteins.^28^ Extensive evidence has shown that UCHL1, a key member of the Ub C‐terminal hydrolases subfamily, plays important roles in nervous system disorders,^29^, ^30^ as well as several types of cancer, including nasopharyngeal carcinoma,^31^ lung cancer,^32^ and breast cancer.^33^ However, its involvement in cartilage differentiation has been rarely reported. Here, we have presented a new understanding of the regulatory mechanism underlying chondrogenesis through the UCHL1/HIF‐1α pathway, demonstrating that activation of UCHL1 promotes chondrogenesis. Considering that UCHL1 has been implicated in HIF‐1α deubiquitination^14^ and the involvement of HIF‐1α in the differentiation process of mesenchymal progenitors into chondrocytes,^8^, ^9^ we initially investigated the role of UCHL1 in rat mesenchymal stem cell chondrogenesis. The expression of UCHL1 increased over time during the chondrogenic differentiation of ASCs and BMSCs, while the chondrogenic marker genes showed a decrease following inhibition of UCHL1 using UCHL1 inhibitor. These findings confirm the ability of UCHL1 to enhance chondrogenic differentiation in ADSCs and BMSCs.

CRISPRa is a straightforward method that allows for the activation of UCHL1 expression from its endogenous genomic locus, in contrast to traditional overexpression approaches. This enables us to study the cis‐acting and nuclear functions of UCHL1 more effectively.^28^ Therefore, our aim is to activate endogenous UCHL1 expression in order to enhance chondrogenesis in ASCs and BMSCs. Several viral delivery methods have been developed for the CRISPRa system. The CRISPR components can be packaged into various viral vectors such as lentivirus,^34^ adenovirus,^35^ adeno‐associated virus^36^ or BV,^37^ and then used for either in vitro transduction or in vivo administration. Although AAV is frequently employed for virus delivery, the size of SpdCas9‐VPR (≈6 kb) exceeds the packaging capacity of AAV. This makes it difficult to achieve efficient delivery into stem cells.^38^ In contrast, baculovirus is capable of efficiently delivering large genetic cargoes (up to 38 kb) into ASCs and BMSCs, with transduction efficiencies exceeding 95%.^37^ Importantly, baculovirus does not replicate or integrate its genome into the chromosomes of transduced cells. This significantly reduces the risk of genotoxicity.^39^ However, one limitation of baculovirus is that it usually allows only transient expression for less than 7 days.^40^ This characteristic might limit its direct applicability in the field of regenerative medicine. Cre is a site‐specific recombinase that can facilitate DNA exchange between short target sequences. Recombination occurs as a circular molecule when the flanking target sites are in direct orientation.^41^ A recent study has shown the efficient use of Cre for extending the duration of transgene expression.^37^ In order to extend the duration of UCHL1 expression, we devised a binary system where the transgene present in the original BV was excised by Cre expressed by a second BV, and then recombined into smaller minicircles. The results showed that the Sa‐UCHL1 group maintained statistically similar levels of UCHL1 expression in ASCs and BMSCs for at least 14 days. One potential drawback of this system is the requirement for two BV vectors: one expressing the loxP‐flanked transgene cassette, and the other carrying the Cre gene. Therefore, the feasibility of a one‐vector system that combines both cassettes into a single BV requires additional investigation.

Various dCas9 variants are derived from different microorganisms, including Sp‐dCas9, Sa‐dCas9 and Nm‐dCas9. Among these variants, Sp‐dCas9 is the most extensively utilized.^42^ Nevertheless, the availability of the protospacer‐adjacent motif (PAM, NGG) across the genome limits the utility of Sp‐dCas9.^43^ Consequently, we conducted a comparative analysis using BV to assess various dCas9‐VPR orthologues. Our findings indicate that Sa‐dCas9‐VPR leads to noteworthy activation of UCHL1 in comparison to Sp‐dCas9‐VPR and Nm‐dCas9‐VPR. The differential effects on UCHL1 activation might arise due to differences in the gRNA scaffold, the PAM sequence's impact on the local chromatin structure within the promoter region of UCHL1, and variations in the dCas9‐VPR protein architecture.

Additionally, we elucidated the molecular mechanism underlying chondrogenesis through UCHL1 promotion. The results of qRT‐PCR and immunofluorescence analysis revealed that UCHL1 does not affect HIF‐1 mRNA levels, but increases the expression of HIF‐1 protein. These findings indicate that UCHL1 safeguards HIF‐1 from degradation by promoting its stabilization. Moreover, in the Sa‐UCHL1 group, HIF‐1 predominantly localized to the nucleus, whereas the other groups exhibited cytoplasmic localization. This suggests that UCHL1 not only stabilizes HIF‐1 but also modulates its cellular distribution. As HIF‐1α is a transcription factor, we identified its target genes (GLUT1,^44^ LDHA,^45^ PGK1^46^) using qRT‐PCR across three groups. The expression levels of these genes were notably higher in the Sa‐UCHL1 group compared to the other two groups, which is consistent with the immunostaining findings. Moreover, the inhibition of HIF‐1α abolished the UCHL1‐induced elevation in sulphated GAG levels. These data demonstrate that HIF‐1α mediates the UCHL1‐promoted chondrogenic differentiation. The findings of this study offer direct evidence that UCHL1 promotes cartilage differentiation in ADSCs through the mediation of HIF‐1α/SOX9. These results provide insights for cartilage regeneration and suggest a novel therapeutic target for treating human cartilage defects.

Nevertheless, certain questions still need to be addressed. Compared to BMSCs, ADSCs are readily isolated in large quantities and possess the ability to differentiate into chondrogenic, osteogenic and adipogenic lineages. As a result, they hold great promise for applications in regenerative medicine.^47^ However, the acquisition of ADSC cells necessitates a secondary operative procedure, which imposes additional trauma on patients and raises corresponding ethical concerns. Induced pluripotent stem cells (iPSCs) can be derived from numerous mature somatic cells,^48^ thereby circumventing the ethical concerns associated with the use of traditional stem cells. CRISPR/Cas9‐mediated genomic editing does not adversely affect the pluripotency and differentiation capacity of iPSCs,^49^ suggesting that CRISPR/Cas9 techniques have the potential to greatly enhance the field of genome editing in iPSC research.

## 5 CONCLUSIONS

**FIGURE 6:**
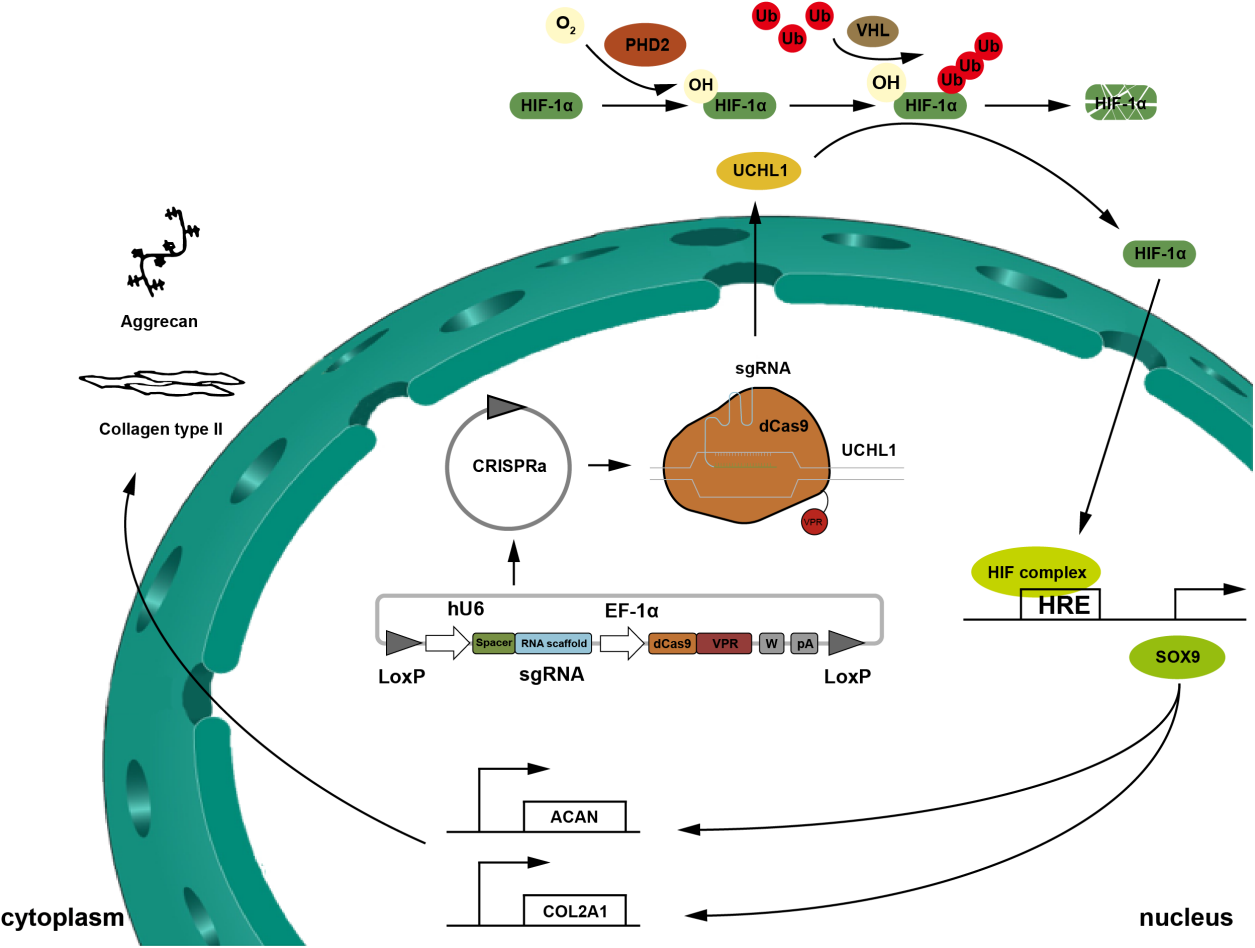
The schematic graph of this study. The stability and localization of HIF‐1α are modulated through CRISPR‐mediated activation of UCHL1 using Sa‐dCas9‐VPR, thereby regulating SOX9‐mediated chondrogenic differentiation.

In summary, our data highlight the ability of CRISPR‐mediated activation of UCHL1 using Sa‐dCas9‐VPR to modulate the stability and localization of HIF‐1α, thereby regulating SOX9‐mediated chondrogenic differentiation (Figure 6). Our findings provide a new avenue for cartilage regeneration and offer potential as a novel therapeutic target for promoting cartilage healing in the future.

## AUTHOR CONTRIBUTIONS

**Shanwei Shi:** Conceptualization (equal); data curation (lead); formal analysis (lead); investigation (lead); methodology (lead); resources (lead); software (lead); visualization (equal); writing – original draft (lead). **Yang Ge:** Writing – review and editing (equal). **Qiqian Yan:** Writing – review and editing (equal). **Shuangquan Wan:** Writing – review and editing (equal). **Mingfei Li:** Writing – review and editing (equal). **Maoquan Li:** Conceptualization (equal); funding acquisition (lead); project administration (lead); supervision (lead); validation (lead); writing – review and editing (lead).

## FUNDING INFORMATION

This study was funded by the Science Research Cultivation Program of Stomatological Hospital, Southern Medical University (no. PY2021028), Medical Science and Technology Research Fund of Guangdong Province (no. B2023405) and Basic and Applied Basic Research Foundation of Guangzhou (no. 2023A04J0427). The funding body played no role in the design of the study and collection, analysis, and interpretation of data and in writing the manuscript.

## CONFLICT OF INTEREST STATEMENT

The authors declare no competing interests.

## Supporting information


Data S1:

## Data Availability

The data that support the findings of this study are available from the corresponding author upon reasonable request.
