# Supplementary material for: Activating UCHL1 through the CRISPR activation system promotes cartilage differentiation mediated by HIF‐1α/SOX9
Source: J Cell Mol Med. 2024 Sep 2;28(17):e70051. doi: 10.1111/jcmm.70051 (PMC11369205; doi:10.1111/jcmm.70051)
Supplement: Supplementary file 1 — Data S1: [file JCMM-28-e70051-s001.docx]

**Supporting Information**

**Supplementary Figures**


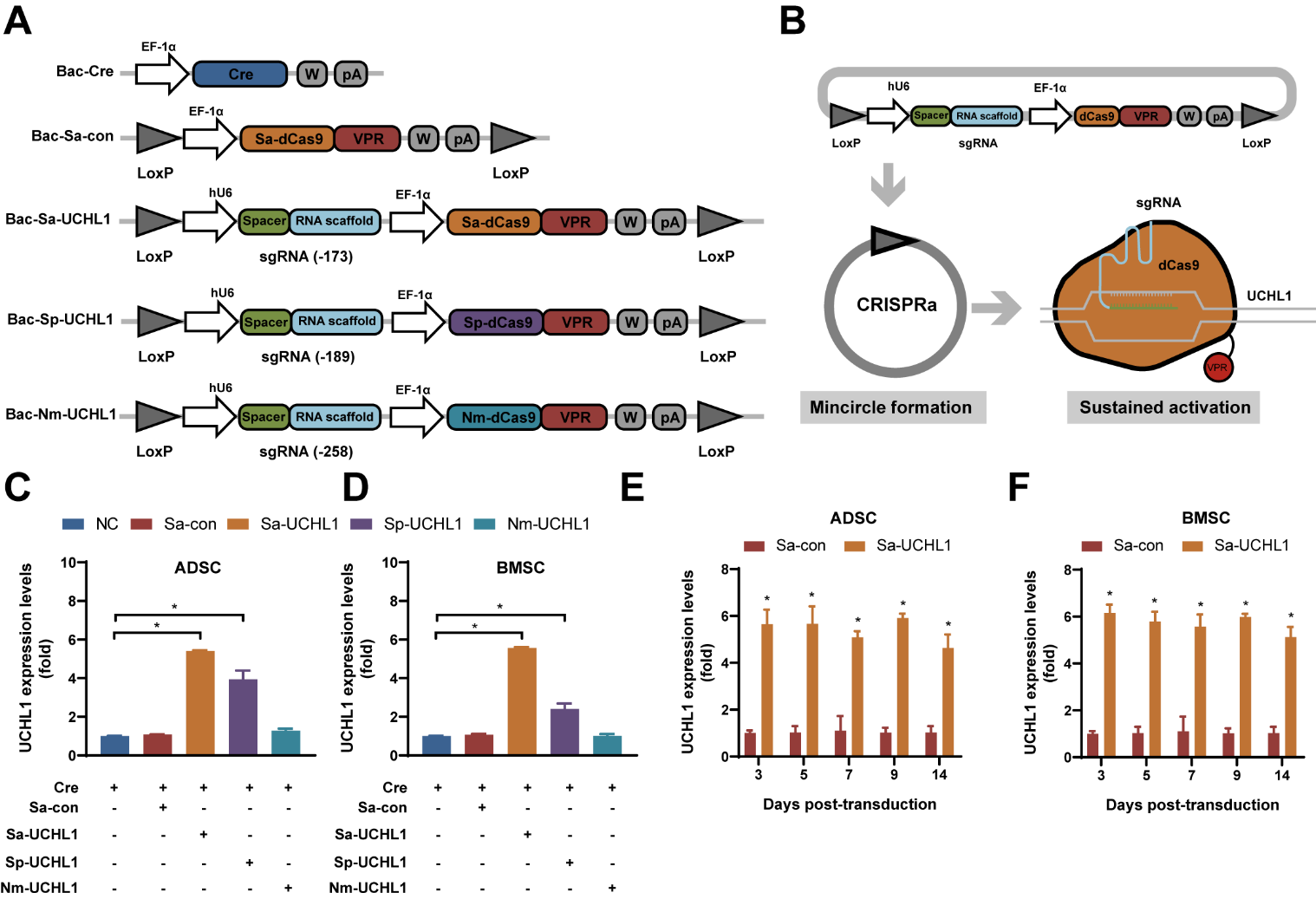


Figure S1. CRISPRa system effectively and persistently activated UCHL1 upregulation.

(A) Schematic illustration of Bac-Cre, Bac-Sa-con, Bac-Sa-UCHL1, Bac-Sp-UCHL1 and Bac-Nm-UCHL1 along with their corresponding gRNAs. The numbers following each gRNA indicate their relative position to the transcription start site. Various dCas9-VPR/gRNA pairs identify distinct PAM sequences upstream of UCHL1 for activation. (B) Concept of BV design. The hybrid BV carries the CRISPRa module expressing sgRNA (comprising spacer and scaffold motif) under hU6 promoter and dCas9-VPR under rat EF-1α promoter. The whole CRISPRa module is flanked by loxP sites, so that Cre recombinase can excise the CRISPRa module off BV genome, forming episomal minicircles. Minicircles can persistently express dCas9-VPR and sgRNA, which orchestrate to bind UCHLU for sustained activation. (C, D) The mRNA expression level of UCHL1 was assessed in ADSCs (C) and BMSCs (D) co-transduced with 3 pairs of baculoviruses at 3 dpt. (E, F) The mRNA expression level of UCHL1 was analyzed in ADSCs (E) and BMSCs (F) co-transduced with either Bac-Cre/Bac-Sa-con or Bac-Cre/Bac-Sa-UCHL1. (Data are presented as means ± SD, Significant differences are presented as **p < 0.05.* W, WPRE sequence. pA, polyadenylation signal.)


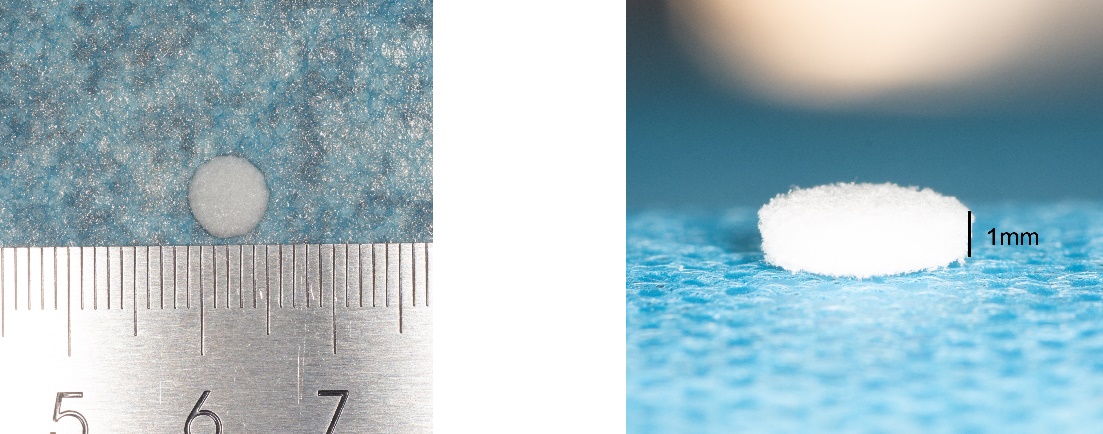


Figure S2 The images of the blank scaffold.


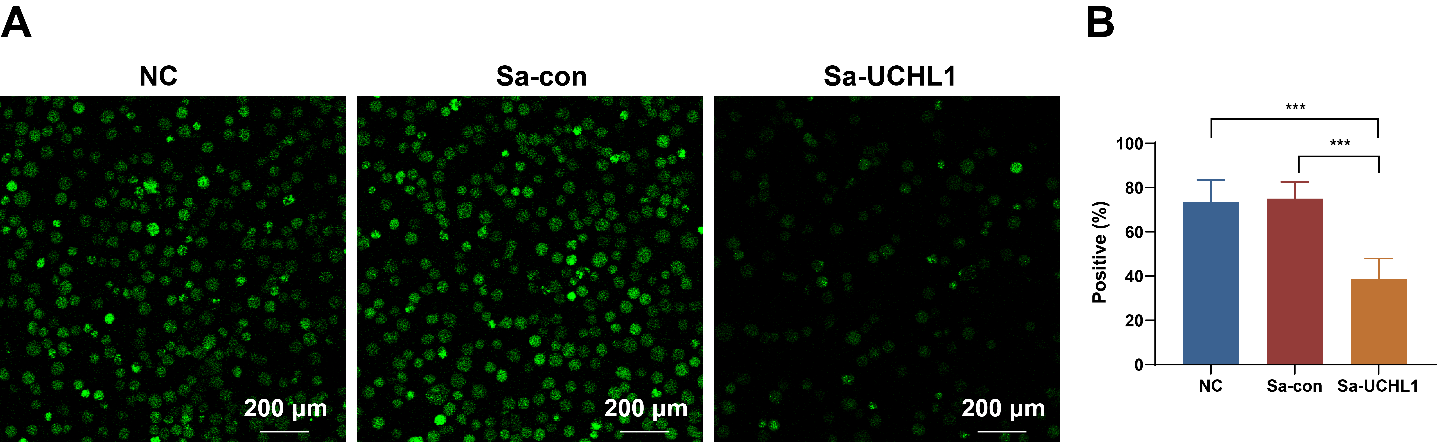


Figure S3 TUNEL staining images (A) and the corresponding quantification (B) in NC, Sa-con and Sa-UCHL1 groups. (NC, negative control, Data are presented as means ± SD, Significant differences are presented as **p < 0.05.)*

**Supplementary Tables**

**Table S1. Primer sequences used for construction of recombinant BVs**

| **Primer ID** | **Primer sequence (5’🡪3’**, enzyme sites are underlined) |
| --- | --- |
| CMV-EF-1α_F (XhoI) | CCCTCGAGACGTTACATAACTTACGG |
| CMV-EF-1α_R (BamHI) | CGCGGATCCTTGCTTTGAATTAGCGGT |
| Cre-WPRE_F (EcoRI) | CCGGAATTCATGTCCAATTTACTGACCGTACACCA |
| Cre-WPRE_R (NotI) | AAGGAAAAAACGCGGCCGCTTGCGGGGAGGCGGCC |
| Sp-dCas9_F (PacI) | GCCTTAATTAAGATGGACAAGAAGTACAGCATCGG |
| Sp-dCas9_R (MluI) | CGACGCGTTGTCGCCTCCCAGCTGAGA |
| Nm-dCas9_F (PalI) | GCCTTAATTAAGATGGCCGCCTTCAAGCCCAAC |
| Nm-dCas9_R (MluI) | CGACGCGTAGCGCACAGGAGGGCGCT |

| **Table S2. Spacer (targeting) sequence for UCHL1** | | |
| --- | --- | --- |
| dCas9 protein | Distance to TSS^a^ | Spacer sequence (5’ 🡪 3’) |
| SadCas9 | -173 | ACCACCAGATTAGCTCACCG GCGAGT |
| SpdCas9 | -189 | CACTCGCCGGTGAGCTAATCTGG |
| NmdCas9 | -258 | GCATGGAGCAGCCTTCTTGA TGGAGCTT |

a TSS: transcription start site.

| **Table S3. Primer sequences used for qRT-PCR** | | |
| --- | --- | --- |
| Gene | Forward | Reverse |
| UCHL1 | TGAAGCAGACCATCGGGAAC | GAGTCATGGGCTGCCTGAAT |
| SOX9 | TCCCCGCAACAGATCTCCTA | AGCTGTGTGTAGACGGGTTG |
| COL2A1 | ATGAGGGCGCGGTAGAGAC | TCACAGACACAGATCCGGCA |
| ACAN | AAGGGCGAGTGGAATGATGT | CGTTTGTAGGTGGTGGCTGTG |
| HIF-1α | AGAGGTTGAGGGACGGAGAT | GCACCAAGCAGGTCATAGGT |
| GLUT1 | GCTGTGGCTGGCTTCTCTAA | CCGGAAGCGATCTCATCGAA |
| LDHA | ATGGCCTGTGCCATCAGTATC | AAGATATCCACTTTGCCAGAGAC |
| PGK1 | GACCGAATCACCGACCTCTC | CAGGATGACAGACCCAGCAG |
| 18S | TTGCCACCCATACACCAGTC | TGGCCTCCTCGGGTGTAATA |
| GAPDH | CCGCATCTTCTTGTGCAGTG | ACCAGCTTCCCATTCTCAGC |

**Table S4. Sequence information of Luciferase reporter assay**

|  | **Sequence (5’🡪3’)** |
| --- | --- |
| Proximal SOX9 promoter sequence for promoter activity assay | AAACTGTGTTTAAGAAACGCCAACGCAGCTGAGGGATACCAGTGAAAAAGCAGATTCTTGCCTTTGTTCCGCTCTCCTGGGCTATACTGCTCGGTAACAATACCTGCATTCAAGCTGTGCCAAGGCAGCACAACGCACGTACCCTACCAGTGTAGATAAGAGTAGGCAGGCAAAATGACTTTCAACAATATTTTGCTTCATGTTAACTGT (-251 ~ -41 from TSS^a^) |
| Hypoxia response element (HRE) | CACGT (complementary strand: ACGTG), -83 ~ -79  from TSS |
| Primers used in promoter cloning | Forward: AAGCTTACTGCTGGTTTAGCTC  Reverse: AGATCTCTGCTCCCAATCCTAC |

a TSS: transcription start site.
